# Adrenal wash-out CT: moderate diagnostic value in distinguishing benign from malignant adrenal masses

**DOI:** 10.1530/EJE-21-0650

**Published:** 2021-11-23

**Authors:** Wiebke Schloetelburg, Ines Ebert, Bernhard Petritsch, Andreas Max Weng, Ulrich Dischinger, Stefan Kircher, Andreas Konrad Buck, Thorsten Alexander Bley, Timo Deutschbein, Martin Fassnacht

**Affiliations:** 1 Department of Radiology, Department of Internal Medicine, University Hospital, University of Würzburg, Würzburg, Germany; 2 Department of Nuclear Medicine, Department of Internal Medicine, University Hospital, University of Würzburg, Würzburg, Germany; 3 Division of Endocrinology and Diabetes, Department of Internal Medicine, University Hospital, University of Würzburg, Würzburg, Germany; 4 Institute of Pathology, University of Würzburg, Würzburg, Germany; 5 Medicover Oldenburg MVZ, Oldenburg, Germany

## Abstract

**Objective:**

Reliable results of wash-out CT in the diagnostic workup of adrenal incidentalomas are scarce. Thus, we evaluated the diagnostic accuracy of delayed wash-out CT and determined thresholds to accurately differentiate adrenal masses.

**Design:**

Retrospective, single-center cohort study including 216 patients with 252 adrenal lesions who underwent delayed wash-out CT. Definitive diagnoses based on histopathology (*n* = 92) or comprehensive follow-up.

**Methods:**

Size, average attenuation values of the adrenal lesions in all CT scan phases, and absolute and relative percentage wash-out (APW/RPW) were determined by an expert radiologist blinded for clinical data. Adrenal lesions with unenhanced attenuation values >10 Hounsfield units (HU) built a subgroup (*n* = 142). Diagnostic accuracy was calculated.

**Results:**

The study group consisted of 171 adenomas, 32 other benign tumors, 11 pheochromocytomas, 9 adrenocortical carcinomas, and 29 other malignant tumors. All (potentially) malignant and 46% of benign lesions showed unenhanced attenuation values >10 HU. In this most relevant subgroup, the established thresholds of 60% for APW and 40% for RPW misclassified 35.9 and 35.2% of the masses, respectively. When we applied optimized cutoffs (APW >83%; RPW >58%) and excluded pheochromocytomas, we missed only one malignant tumor by APW and none by RPW. However, only 11 and 15% of the benign tumors were correctly identified.

**Conclusions:**

Wash-out CT with the established thresholds for APW and RPW is insufficient to reliably diagnose adrenal masses. Using the proposed cutoff of 58% for RPW, malignant tumors will be correctly identified, but the added value is limited, namely 15% of patients with benign tumors can be prevented from additional imaging or even unnecessary surgery.

## Introduction

Adrenal lesions are routinely encountered in about 3–5% of the patients undergoing abdominal CT (1, 2, 3, 4). In most cases, imaging is performed for reasons other than for suspected adrenal disorders, and these masses are commonly referred to as adrenal incidentalomas (5). Although the vast majority of these lesions is benign and endocrine inactive, up to 10% are malignant and about 10% show a clinically relevant hormone excess (3, 6), both conditions that usually require surgery (7).

Earlier recommendations suggested follow-up imaging at two to four subsequent occasions in all adrenal masses without indication for surgery (8, 9, 10). However, this approach leads to significant costs, radiation exposure, and psychological burden for the patients. In 2016, the clinical practice guidelines of the European Society of Endocrinology together with the European Network for the Study of Adrenal Tumors (ENSAT) recommended a detailed work-up at the time of first presentation to achieve a definitive diagnosis and to avoid unnecessary follow-up imaging (7).

Several imaging modalities have been proposed to differentiate benign from malignant adrenal lesions, but so far only unenhanced CT has gained general acceptance (5, 7, 11). In patients without history of extra-adrenal malignant diseases, adrenal masses with CT attenuation ≤10 Hounsfield units (HU) virtually always represent lipid-rich benign adenomas or other benign tumors (12, 13). However, if the adrenal mass is detected during staging of a malignant disease, tumor attenuation ≤10 HU appears less suitable for ruling out malignancy (12). A major challenge is that at least 30% of adrenal adenomas contain only small amounts of intracellular fat resulting in higher unenhanced attenuation. Recently, the UrineACT study suggested to increase the cutoff value to 20 HU (14). Of note, however, some malignant tumors would be missed by this approach, and therefore, this cutoff is not yet generally accepted.

Since the first description of an adrenal wash-out CT in 1998 (15), this method has repeatedly been reported for the determination of benign or malignant adrenal lesions (16, 17, 19). In brief, the idea is that the absolute or relative percentage wash-out (APW/RPW) of the contrast agent 10–15 min after its application is high in benign and low in malignant adrenal lesions. However, most studies evaluating the accuracy of delayed wash-out CT carried a high risk of bias and were ineligible for a meta-analysis published in 2016 (e.g. due to lack of an appropriate reference standard, small size of series, and poor reporting of the clinical indication for CT imaging) (12). In a recent French study including 82 adrenal masses, the authors suggested that cutoffs for delayed wash-out CT for APW/RPW of 60 or 40% were accurate in patients with low suspicion for a malignant disease (e.g. excluding imaging for staging of malignant diseases) (18). The fact that this series is the largest with well-established reference standard so far clearly indicates the weak evidence in this context.

Therefore, the aim of this study was to re-evaluate the diagnostic accuracy of wash-out CT (in a large well-balanced study population with a clearly defined robust reference standard) and to determine thresholds allowing for a reliable diagnosis of a benign adrenal mass.

## Patients and methods

### Patients

**Figure 1:**
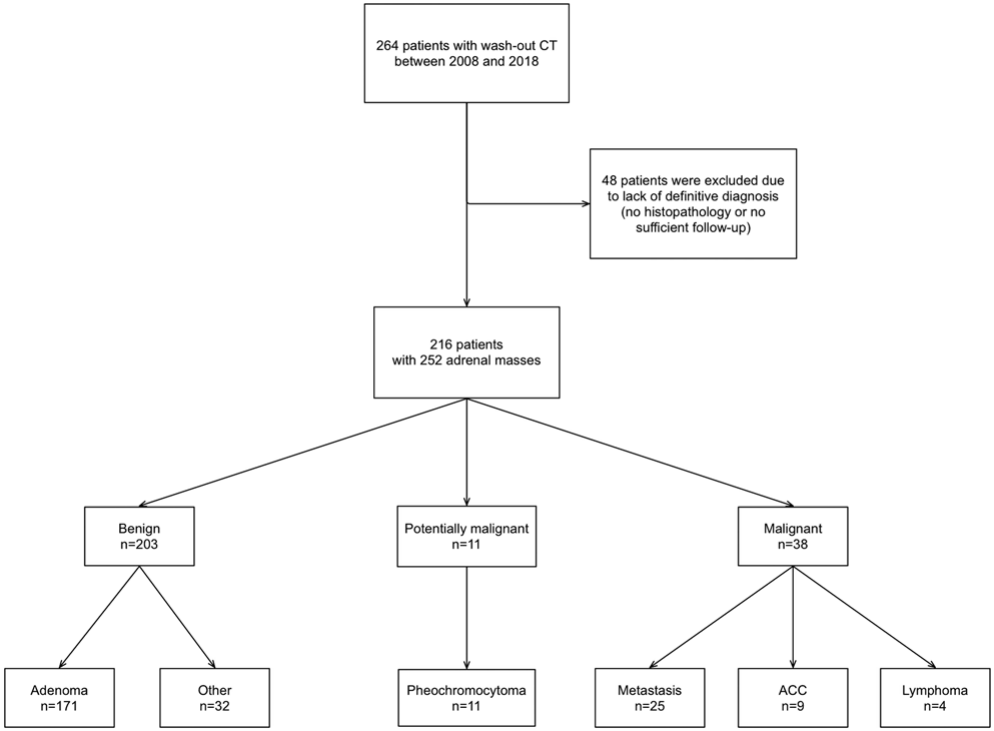
Flow diagram of patients investigated in this study. ACC, adrenocortical carcinoma.

Our hospital performs annually about 7000 abdominal CT scans, of which in about 4–5% adrenal lesions are detected. For this study, we evaluated all wash-out CT scans (performed between January 2008 and December 2018) of patients with adrenal masses managed at the Würzburg University Hospital. After exclusion of procedures not related to adrenal tumors (defined as any mass ≥1 cm), we identified 264 patients. We defined upfront criteria to classify whether an adrenal tumor is benign or malignant (Supplementary Table 1, see section on supplementary materials given at the end of this article). For cases with insufficient data, for example incomplete medical history or hormonal work-up or missing follow-up imaging, follow-up examinations were scheduled. Forty-eight patients had to be excluded due to insufficient information to allow a definitive diagnosis (Fig. 1).

Our final study cohort consisted of 216 patients with 252 adrenal lesions (including 36 bilateral lesions) (Table 1). Of these, 47 (19%) were detected during tumor evaluation due to an extra-adrenal malignancy. We evaluated the demographics, indications for imaging, size and internal structure of the adrenal mass, unenhanced and wash-out attenuation values, all follow-up imaging examinations, hormonal work-up, previous or concomitant malignancies, and, if available, histopathological findings. Tumors were judged as benign either by histology (*n* = 61), absence of significant progression during follow-up imaging (*n* = 124), or by missing clinical evidence of malignant disease of at least 5 years clinical follow-up (*n* = 18) (details see Supplementary Table 1). Diagnosis of malignant tumors was based on histology (*n* =31), significant change in tumor size during follow-up (*n* = 14) or in case of an obviously new adrenal mass that fulfilled additional criteria (e.g. size >2 cm, evidence of an extra-adrenal malignancy) (*n* = 5) (Supplementary Table 1).

**Table 1:** Baseline characteristics of all patients and adrenal masses.

	Entire cohort	Subgroup*
Patients, *n*	216	128
Sex, *n* (%)		
Male	118 (55)	73 (57)
Median age (range)	60 (30–83)	59 (30–83)
Mode of detection of the adrenal mass (%)		
Incidental finding	174 (69)	96 (68)
Suspected adrenal disease	31 (12)	14 (10)
Tumor evaluation due to an extra-adrenal malignancy	47 (19)	32 (23)
Side of adrenal mass, *n*		
Right	65 (30.1)	44 (34.4)
Left	115 (53.2)	70 (54.7)
Bilateral	36 (16.7)	14 (10.3)
Total number of adrenal masses	252	142
Final diagnosis, *n* ^1^		
Benign	203	93
Adrenal adenoma/ hyperplasia	171^2^	75^3^
Other benign tumor	32^4^	18^5^
Malignant	38	38
Metastasis	25^6^	25^6^
Adrenocortical carcinoma	9^7^	9^7^
Lymphoma	4^8^	4^8^
Potentially malignant	11	11
Pheochromocytoma	11^7^	11^7^

^1^For definition, see Supplementary Table 1; ^2^50 confirmed by histology; ^3^29 confirmed by histology; ^4^11 confirmed by histology; including histological-confirmed atypical myelolipomas (*n* = 6), atypical adrenal cysts (*n* = 2), ganglioneuromas (*n* = 2), tuberculosis-related adrenal masses (*n* = 1); ^5^5 confirmed by histology; ^6^including primaries from urogenital system (*n* = 6), skin (*n* = 5), lung (*n* = 4), gastrointestinal system (*n* = 4), breast (*n* = 1), leiomyosarcoma (*n* = 2), liver (*n* = 1), breast (*n* = 1), CUP (*n* = 1), etc.; among them, 10 were confirmed by histology; ^7^all confirmed by histology; ^8^1 confirmed by histology. *Subgroup with adrenal mass HU >10 in unenhanced CT.

This retrospective single-center study was conducted in accordance with the principles of the Declaration of Helsinki. The majority of the patients (*n* = 161) were included in the ENSAT registry, which is approved by the ethics committee of the University of Wuerzburg (No. 88/11), and these patients have signed the informed consent. For other patients (*n* = 55), the ethics committee waived the requirement of informed consent as the data were collected under conditions of regular clinical care.

### CT data acquisition

Over a 10-year study period, technical improvements entailed that different CT scanners (16-row, 64-row, and dual-source) had been used for image acquisitions. Regardless of the scanner technology used, the scan protocol included an unenhanced CT scan, followed by a bolus-triggered dynamic contrast-enhanced protocol with scans in the arterial phase, venous phase (60 s after bolus tracking), and a delayed phase 10–15 min after injection of the contrast agent.

### CT image analysis

All images were re-evaluated by a board-certified radiologist (W.S.) blinded to all clinical data. The size of the adrenal mass and changes in size during follow-up were measured adapting the Response Evaluation Criteria in Solid Tumors (version 1.1) by using the trans-axial slice with the largest tumor diameter.

Following radiological standards (12, 20), we initially planned to exclude tumors with heterogeneous internal structure. However, judgment of the internal structure of an adrenal lesion (especially if it is small) is challenging and comes with a high interobserver variability. Furthermore, we realized that 31% of our samples were heterogeneous and would have to be excluded (Table 2). Therefore, we decided to keep these samples and perform a subanalysis on this topic. Average attenuation values were measured in HU with a circular region of interest (ROI) tool placed in the center and by including at least two-third of the lesion, carefully recessing the lesion’s margins to minimize partial volume effects. The attenuation values of the lesion in the unenhanced scan and the following contrast-enhanced scans were measured on the corresponding slice at precisely the same level (matched slices). In larger heterogeneous adrenal masses, the ROI was drawn within the most homogenous area in the venous/portalvenous phase and then transferred to other phases. Calcifications and cystic and necrotic areas within the lesion were excluded. To calculate the APW and RPW, we used the following formulas:

**Table 2:** Characteristics of all adrenal masses (all adrenal lesions, *n*  = 252) under study and of the subgroup of adrenal masses with HU >10 in unenhanced CT. Values are presented as median (range).

	Adenoma	Other benign lesion	Pheochromocytoma	Metastasis	Adrenocortical carcinoma	Lymphoma
All adrenal masses						
*n* (% of total)	171 (68)	32 (13)	11 (4)	25 (10)	9 (4)	4 (2)
Size (in mm) mean (range)	26 (10 to 112)	60 (10 to 218)	53 (20 to 128)	39 (11 to 98)	63 (17 to 116)	74 (52 to 110)
Percentage of samples with homogenous internal structure	70.8	46.8	36.4	80	77.8	100
Attenuation values in HU						
Unenhanced	8.2 (−19 to +78)	14.1 (−98 to +65)	29.7 (14 to 44)	31.9 (19 to 41)	30.9 (22 to 40)	31.4 (30 to 10)
Arterial phase	31.1 (−6 to +111)	30.7 (−93 to +114)	53.8 (18 to 212)	42.6 (26 to 116)	52.1 (26 to 68)	41.5 (37 to 56)
Venous phase	53.9 (−1 to +120)	39 (−87 to +80)	61 (16 to 111)	55 (32 to 110)	75 (25 to134)	58.5 (55 to 67)
Delayed phase	23.3 (−14 to +84)	31.8 (−86 to +70)	46.6 (15 to 79)	50.7 (27 to 66)	52.6 (25 to 87)	54.1 (50 to 65)
Percentage of wash-out						
APW	69.2 (0 to 99)	12 (0 to 85)	47.1 (0 to 83)	43.5 (0 to 114)	51.9 (8 to 71)	21.5 (0 to 32)
RPW	59 (0 to 261)	0 (−45 to 66)	15.5 (0 to 70)	15.8 (0 to 58)	27.3 (3 to 38)	9.3 (0 to 15)
Subgroup analysis						
*n* (% of total )	75 (53)	18 (13)	11 (8)	25 (18)	9 (6)	4 (3)
Size (mm) mean (range)	29 (10 to 96)	43 (11 to 105)	54 (21 to 128)	41 (15 to 98)	65 (17 to 116)	74 (52 to 110)
Percentage of samples with homogenous internal structure	65.3	61.1	36.4	80	77.8	100
Attenuation values in HU						
Unenhanced	24.4 (11 to 79)	27.3 (10 to 66)	29.7 (14 to 44)	31.9 (19 to 41)	30.9 (22 to 40)	31.4 (30 to 10)
Arterial phase	46.9 (15 to 111)	37.9 (12 to 114)	53.8 (18 to 212)	41.9 (26 to 115)	52.1 (26 to 68)	41.5 (36 to 55)
Venous phase	66.2 (16 to 120)	48 (14 to 80)	61 (16 to 112)	54.7 (32 to 110)	75 (25 to 134)	58.5 (56 to 67)
Delayed phase	40 (11 to 84)	40.9 (13 to 70)	46.6 (15 to 79)	55.5 (27 to 66)	52.2 (25 to 87)	54 (50 to 54)
Percentage of wash-out						
APW	67.7 (0 to 99)	21 (0 to 85)	47.1 (0 to 83)	43.5 (0 to 114)	52 (8 to 71)	21.5 (0 to 32)
RPW	43.9 (0 to 80)	5.6 (0 to 58)	15.5 (0 to 70)	15.8 (0 to 58)	27.3 (3 to 38)	9.3 (0 to 15)

APW, absolute percentage wash-out; RPW, relative percentage wash-out.


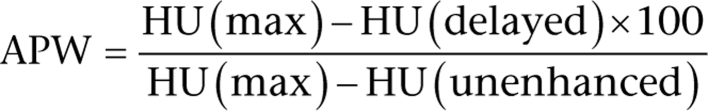



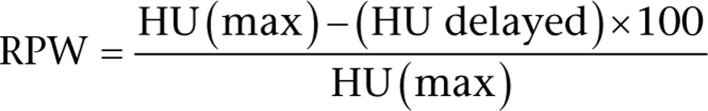


We adjusted the usual formulas replacing HU (venous) with HU (max) due to the following reason: The usual formulas require that lesions show the highest attenuation values in the venous phase; however, this is not always the case. For example, some lesions show a slow ‘wash-in’ phenomenon leading to a maximum of attenuation in the delayed phase, resulting in negative wash-out values. Our adjusted formulas provide unchanged values for lesions with typical contrast dynamic and equal zero for lesions that do not show a wash-out.

### Statistical analysis

For statistical analysis, IBM SPSS Version 26 was used. Data were reported as mean ± s.d. or median and range, as appropriate. As adrenal lesions with unenhanced attenuation values ≤10 HU are generally accepted as benign lipid-rich adenomas (), a subgroup containing all lesions with unenhanced attenuation values >10 HU was built. The target condition was defined as any etiological diagnosis that would not necessitate surgery or further imaging exploration, that is adenomas and other benign tumors. In contrast, the group of (potentially) malignant tumors comprised adrenocortical carcinomas, metastases of extra-adrenal malignancies, adrenal lymphomas, and pheochromocytomas. The latter tumor entity was included here because it is well known that they cannot be discriminated by conventional imaging criteria and pathologists cannot judge them as unequivocally benign. The diagnostic accuracy of size, unenhanced CT attenuation (HU), RPW, and APW were estimated for the total group as well as for the subgroup. For each parameter, sensitivity, specificity, and positive and negative predictive values for the detection of benign masses were estimated, and 95% CI were calculated by using the exact binomial distribution for proportions. Sensitivity was defined as the probability to have a positive test in patients with the target condition. Specificity was defined as the probability to have a negative test in patients without the target condition. In the first step, we used the published cutoffs for RPW and APW of >40 and 60%, respectively (12, 18, 21, 22, 23) as indicators for benign lesions. Receiver-operating characteristic (ROC) curves were created for APW and RPW. The area under the curve (AUC) and its CI was estimated by using the % ROC macro. Based on those, we defined upfront aiming at alternative thresholds to receive specificity values ≥97%. A non-parametric test (Mann–Whitney *U* test) was used to determine the significance of differences between the mean APW and RPW for benign and malignant adrenal lesions. Significance was set at *P*  < 0.05.

## Results

Key characteristics of patients and tumors (including the subgroup of lesions with unenhanced attenuation values >10 HU) are shown in Tables 1 and  2. Detailed test categories are indicated in Tables 3 and 4 (and Supplementary Tables 2 and 3). The following sections refer either to the entire group of 252 adrenal masses or to the subgroup of lesions with unenhanced HU >10 (*n* = 142). In addition, we provide a subanalysis of patients in whom the tumor was detected incidentally (and not as part of an tumor evaluation or due to an suspected adrenal disease) in Supplementary Table 4a and b.

**Table 3:** Performance of tests in the entire cohort. To make this table easier to read, 95% CIs were omitted. These are provided in Supplementary Table 2.

Test categories	Cutoff	Benign (*n* = 203)	(Potentially) malignant (*n* = 49)	% of benign cases	% of (potentially) malignant cases	PPV %	NPV %
Tumor size							
	<4 cm	159	22	78.3^1^	44.9	87.9	38.0
	≥4 cm	44	27	21.7	55.1^2^		
Unenhanced Hounsfield Units							
	≤10	110	0	54.2^1^	0	100	34.5
	>10	93	49	45.8	100^2^		
Absolute percentage wash-out							
	>60%	130	11	64.0^1^	22.4	92.2	34.2
	≤60%	73	38	36.0	77.6^2^		
	>83%	17	1	8.4^1^	2.0	94.4	20.5
	≤83%	186	48	91.6	98.0^2^		
Relative percentage wash-out							
	>40%	140	4	69.0^1^	8.2	97.2	41.7
	40%	63	45	31.0	91.8^2^		
	>58%	93	1	45.8^1^	2.0	98.9	30.4
	≤58%	110	48	54.2	98.0^2^		

^1^Sensitivity; ^2^Specificity. PPV, positive-predictive value; NPV, negative-predictive value

**Table 4:** Performance of tests in the subgroup of adrenal masses with unenhanced HU >10.

Test categories	Cutoff	Benign (*n* = 93)	(Potentially) malignant (*n* = 49)	% of benign cases	% of (potentially) malignant cases	PPV %	NPV %
Tumor size							
	<4 cm	70	22	75.3^1^	44.9	76.1	54.0
	≥4 cm	23	27	24.7	55.1^2^		
Absolute percentage wash-out							
	>60%	53	11	57.0^1^	22.4	82.8	48.7
	≤60%	40	38	43.0	77.6^2^		
	>83%	10	1	10.8^1^	2.0	90.9	36.6
	≤83%	83	48	89.2	98.0^2^		
Without pheos (*n* = 131)	>83%	10	1	10.8^1^	2.6	90.9	30.8
	≤83%	83	37	89.2	97.42		
Relative percentage wash-out							
	>40%	47	4	50.5^1^	8.2	92.2	49.5
	≤40%	46	45	49.5	91.8^2^		
	>58%	14	1	15.1^1^	2.0	93.3	37.8
	≤58%	79	48	84.9	98.0^2^		
Without pheos (*n* = 131)	>58%	14	0	15.1^1^	0	100	32.5
	≤58%	79	38	84.9	1002		

^1^Sensitivity; ^2^Specificity; Pheos, pheochromocytomas. To make this table easier to read, 95% CIs were omitted. These are provided in Supplementary Table 3.

### Tumor size and morphologic criteria of benignity

The mean size of all adrenal masses was 3.5 cm (±2.7) with a range from 1 to 21.8 cm. A size of ≥4 cm was reported in 22% of all benign and 55% of all (potentially) malignant lesions. The ROC curve for size for all lesions and for the subgroup had an AUC of 0.71 (95% CI 0.63–0.79) and 0.70 (0.60–0.79), respectively. In addition, we performed an analysis of ‘classical benign imaging features’, namely homogenous internal structure, sharply delineated and harmonic rounded-oval form. Of note, only 62.5% of the adenomas fulfilled all these criteria, whereas this was the case also for 66 and 68% of the adrenocortical carcinomas (ACCs) and metastases in our series.

### Unenhanced CT

All (potentially) malignant and 46% of all benign lesions presented >10 HU in unenhanced CT. The ROC analysis for unenhanced CT revealed the AUC of 0.89 (95% CI 0.85–0.93) for all lesions and of 0.76 (0.68–0.84) for the subgroup. At a threshold of ≤10 HU, the sensitivity, specificity, PPV, and NPV to diagnose a benign lesion were 54 (47–61), 100 (93–100), 100 (100–100), and 35% (31–38), respectively. Using a cutoff of ≤20 HU, these values were 73 (66–79), 95.9 (86–100), 99 (95–100), and 46 (40–52), respectively, and two (potentially) malignant tumors (one metastasis of a lung cancer and one pheochromocytoma) were missed.

### Absolute percentage wash-out

**Figure 2:**
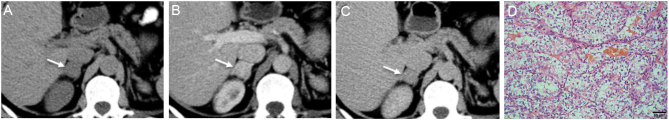
Example of misdiagnosed adrenal metastasis of a renal cell cancer. (A) Unenhanced CT, (B) CT scan in the venous phase, (C) CT scan 15 min after contrast agent application, (D) hematoxylin and eosin stain: APW of 78.3% and RWP of 57.7% suggested a benign tumor, but histology revealed a metastasis of renal cell cancer.

**Figure 3:**
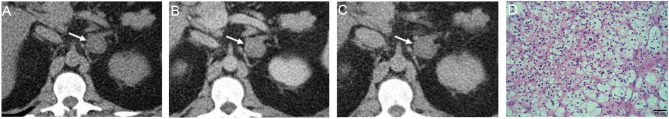
Example of misdiagnosed adrenocortical adenoma. (A) Unenhanced CT, (B) CT scan in the venous phase, (C) CT scan 15 min after contrast agent application, (D) hematoxylin and eosin stain: APW of 20.7% and RPW of 38.5% suggested a malignant tumor, but histology proved an adenoma.

**Figure 4:**
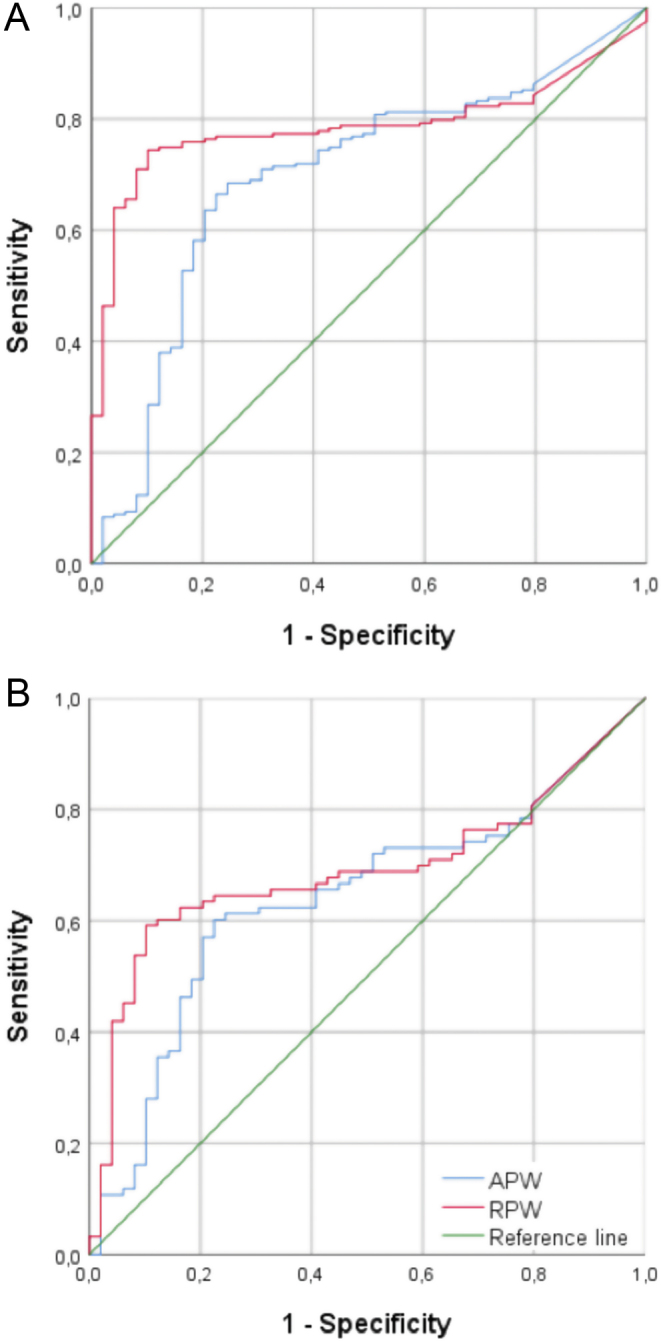
Receiver-operating characteristic curves for absolute (blue curve) and relative (red curve) percentage wash-out (APW and RPW) in the entire cohort of 252 adrenal masses (A) and the subgroup of 142 adrenal masses with unenhanced HU >10 (B). The angle bisector (green line) in both curves represents area under the curve (AUC) = 50. AUC for APW is 0.69 (95% CI: 0.61–0.78) for the entire cohort and 0.64 (95% CI: 0.55–0.73) for the subgroup, whereas the AUC for RPW is 0.79 (95% CI: 0.73–0.84) for all adrenal masses and 0.69 (95% CI: 0.60–0.78) for the subgroup.

The mean APW for all benign lesions was higher than for that malignant lesions: 56 ± 28% (range: 0–99%) vs 38 ± 29% (range 0–114%), respectively (*P*  < .01). Twenty-two percent of all (potentially) malignant lesions showed an APW above 60%, pretending that they were benign, although they were not (Fig. 2). On the other hand, 36% of all benign lesions and 43% of benign lesions in the subgroup presented an APW lower than 60%, falsely indicating that they were malignant (Fig. 3). The AUC for APW was 0.69 (95% CI 0.61–0.78) (Fig. 4). Regarding all lesions, a 60% threshold had a sensitivity, specificity, PPV, and NPV for a benign diagnosis of 64, 78, 92, and 34%, respectively. In the subgroup, the respective values were 57, 78, 83, and 49%, respectively.

### Relative percentage wash-out

The mean RPW for all benign lesions was higher than that for malignant lesions: 50 ± 34% (range: –45 to 261) vs 18 ± 16% (range: 0 to 70), respectively. Sixty-nine percent of all benign lesions (and 50% of the benign lesions in the subgroup) showed an RPW above 40%, whereas in 8% of all (potentially) malignant lesions (one pheochromocytoma and three metastases), the RPW showed results higher than 40% (Fig. 2). The ROC analysis indicated an AUC for RPW of 0.79 (95% CI: 0.73–0.84) (Fig. 4). Regarding all lesions, the 40% threshold led to a sensitivity, specificity, PPV, and NPV for a benign diagnosis of 69, 92, 97, and 42%, respectively. In the subgroup, it resulted in 51, 92, 92, and 50%, respectively.

### Additional analyses

Due to the fact that only two-third of our samples were judged as homogenous, we performed a subanalysis in this cohort. Interestingly, the performance of different tests (Supplementary Table 5) was quite similar to the entire cohort.

Of note, 38 of 252 adrenal masses showed a ‘wash-in’ phenomenon with the highest attenuation value at the delayed scan. These included not only 11 adenomas, 5 myelolipomas, and 10 other benign tumors but also 13 (potentially) malignant tumors (7 metastases, 3 pheochromocytomas, 2 adrenocortical carcinomas, and 1 lymphoma).

From a clinical perspective, misdiagnosing a malignant tumor as benign appears more critical than the other way around. Therefore, we used the ROC curve analysis to determine a threshold to detect at least 97% of all malignancies (specificity for benign tumors ≥97%). With this goal, the optimized cutoff for APW was >83% and for RPW >58% (Tables 3 and  4). When pheochromocytomas were excluded, this method allowed for a specificity of 100%. However, this yielded in low sensitivity (e.g. for APW in the subgroup of only 11%, meaning only 10 of 93 benign lesions were correctly identified). Results for RPW are again slightly better with a sensitivity of 15%.

## Discussion

This largest study on wash-out CT in patients with 252 well-characterized adrenal masses and reliably determined diagnosis indicates that delayed wash-out CT (despite widespread use) cannot be recommended as a standard method to determine whether an adrenal mass is benign or malignant. Using the widely used established thresholds of 40% for RPW or 60% for APW, in our study, 4 and even 11 of 49 (potentially) malignant adrenal tumors were misdiagnosed. Furthermore, only 69 and 64% of benign tumors were classified correctly by RPW and APW, respectively. These numbers are even lower, when focusing only on tumors with HU >10 in unenhanced CT, which are clinically the most relevant subgroup. However, when applying a threshold of >58% for RPW and excluding pheochromocytomas, this method is able to exclude malignancy in all patients, but we have to acknowledge that only 15% of benign tumors are appropriately assigned.

The accurate and efficient characterization of adrenal masses by non-invasive imaging is the most crucial part of the algorithm to assess the risk of malignancy (5, 11, 12, 23, 24). Size of an adrenal lesion is an easily measurable factor and increased size correlates with a higher likelihood of malignancy (1, 25). Not surprisingly, also in our study, benign tumors tended to be smaller, but there is significant overlap (especially with metastases, but also with adrenocortical carcinomas). Furthermore, more than 20% of benign lesions measured more than 4 cm, whereas 45% of unequivocal malignant lesions were <4 cm (including one adrenocortical carcinoma with only 1.7 cm). However, early recognition of malignancy is particularly important in adrenocortical carcinoma since only localized tumors come with the chance of cure by complete resection (12, 26, 27).

Our study confirms that a cutoff of ≤10 HU on unenhanced CT is a reliable tool to identify lipid-rich adenomas. All 110 lesions with unenhanced attenuation values ≤10 HU were benign, resulting in a specificity and PPV of 100%. Although this cutoff seems well established, studies with robust reference standards are still scarce (13). In a recent study, Bancos *et al.* suggested that a cutoff of 20 HU should replace the cutoff of 10 HU for exclusion of adrenocortical carcinoma (14). Although few adrenocortical carcinomas have HU between 10 and 20 (14, 28), this cutoff would have worked in all nine adrenocortical carcinomas of our series. Since another 38 benign adrenal lesions with attenuation values between 10 and 20 HU on unenhanced CT would have been correctly classified, the sensitivity to diagnose benign lesions increases significantly from 54 to 73%. However, we would have missed one pheochromocytoma and one metastasis with this threshold.

For many years, delayed wash-out CT has been propagated as a reliable method to identify lipid-poor adenomas (15). However, a comprehensive systematic meta-analysis analyzing only studies with adequate histological or defined imaging-based follow-up as reference standard could only include two studies with wash-out CT (29, 30) and a total number of 90 adrenal lesions (13). Whereas this method seemed to be promising in patients without history of malignancy, it performed rather poor in adrenal masses detected during evaluation of cancer patients with a higher probability of adrenal metastases. The overall conclusion of the authors was that there is insufficient evidence to rely on this method. In 2018, the largest series to date with wash-out CT (*n* = 82) was published, and the authors concluded that the cutoffs of 40 and 60% for RPW and APW, respectively, are safe, when pheochromocytomas were excluded (19). However, their series included only three patients with metastasis and is, therefore, only applicable for patients without any history of cancer. Of note, to reach 100% sensitivity for all patients (including the pheochromocytomas), this study suggested similar cutoff values as our analysis, namely 53 and 78%, respectively. Our study included 47 tumors detected during evaluation due to an extra-adrenal malignancy and is, therefore, more representative of a cohort with patients with adrenal lesions. However, when focusing only on true incidentalomas (Supplementary Table 3), the results do not differ in a relevant manner.

In a study focusing on CT characteristics of pheochromocytoma, all these tumors (*n* = 374) showed unenhanced attenuation values ≥10 HU, and the authors concluded that biochemical testing for pheochromocytoma is not necessary in adrenal lesions showing unenhanced attenuation values <10 HU (31). In our series, we can confirm that all our pheochromocytomas presented unenhanced attenuation values >10 HU. A recent series from China with 116 adrenal masses (including 63 with unenhanced HU >10) suggested a short-time (200 s) delay wash-out CT (32). Although the authors claimed that this approach could help in differentiating adenomas from non-adenomas, there was significant overlap between groups and at least 10% of the non-adenoma group were misdiagnosed.

The results of our study are less promising as suggested by others, the accuracy of delayed wash-out CT is obviously just moderate. To date, however, none of the currently available methods is able to reliably differentiate lipid-poor adenomas from metastases, lymphomas, or adrenocortical carcinomas, thereby excluding malignancy. A recent study demonstrated that also ^18^F-FDG-PET/CT has significant flaws (33). By analyzing 117 well-characterized adrenal masses with unenhanced HU >20, 10% of benign lesions were FDG-PET positive and 15% of malignant lesions were FDG-PET negative (including 3 adrenocortical carcinomas). The main advantage of wash-out CT is that it can be easily combined with an unenhanced CT, which is undoubtedly the method of choice. Therefore, we have implemented the following diagnostic flow in our hospital: measurement of HU will be done immediately after detection of the adrenal tumor in unenhanced CT. If the value is ≤10 HU, no further imaging is required for this adrenal mass. If the value is >10 HU, the patient will receive contrast agent during the same examination and a delayed wash-out analysis after 1015 min. Applying the RPW cutoff of 58% from this study, we can diagnose lipid-poor adenomas in about 15% of patients, which then do not require any further imaging work-up. We acknowledge that this is a small number, but for these individuals we can avoid a follow-up examination, which comes also with psychological burden for the patients and additional costs for the health system. All other patients will be discussed in the multidisciplinary tumor board and either scheduled for surgery, FDG-PET/CT or – in few patients – follow-up imaging based on individualized approach appreciating the results of the endocrine work-up and desire of the patient.

Although not the primary focus, our study provided some interesting results on the value of ‘classical benign imaging features’ like homogeneity of an adrenal mass, which are by some authors judged as a prerequisite to measure unenhanced CT attenuation or to perform wash-out studies. However, as demonstrated in Table 2 and Supplementary Table 5, homogeneity of an adrenal tumor is on one hand not really helpful in identifying benign lesions, and on the other hand, it has no relevant impact on the test performance of APW and RPW, respectively.

Our study has several limitations. First, there are inherent problems due to its retrospective nature. Second, the CT images were obtained at different CT scanners with variability among equipment, contrast dosing, and timing of scan phases over a long-study period. However, this can also be seen as an advantage because it reflects closer a real-world scenario. Furthermore, the variability of the readers was significantly reduced by re-analyzing all imaging by one experienced radiologist, who was blinded for all clinical, pathological, and follow-up data (including the final diagnosis). Third, our cohort is not representative for all adrenal tumors or incidentalomas because tumors with clear benign features (e.g. unenhanced HU ≤10) were frequently not scheduled for a delayed wash-out CT. On the other hand, in these tumors, wash-out CT is not necessary anyway and one might even argue that this comes with an unnecessary radiation dose. Fourth, although our study with 252 well-characterized adrenal masses is still rather small it is almost as large as all studies with reliable reference standards together. Another strength of this study is the well-balanced composition of our study cohort (including a sufficient number of metastases, but also adrenocortical carcinomas) and the high number of histology-confirmed tumors (Table 1). Furthermore, the reliability of the final diagnosis in patients without histology is quite high. More than 85% of the benign adrenal tumors were followed-up by imaging for at least 12 months. The few remaining masses had either a stable tumor size for more than 6 months plus a negative FDG-PET (*n* = 3) or the patients developed no clinical signs of a malignant disease at least 5 years after the diagnosis of the adrenal tumor (*n* = 18) (details see Supplementary Table).

In conclusion, our well-balanced large study cohort of 252 adrenal masses with a robust reference standard demonstrated the limited value of the established thresholds for APW and RPW to correctly identify malignant adrenal lesions, whereas we could confirm that unenhanced CT can safely identify lipid-rich adrenal adenomas. However, delayed contrast wash-out CT with a cutoff of 58% for RPW may – when applied as part of a multidisciplinary work-up – help to identify some additional patients with benign adrenal mass that do not require additional imaging procedures and follow-up or even prevent unnecessary surgery.

## Supplementary Material

Supplementary Table 1. Criteria to categorize the different adrenal masses and numbers per groups.

Supplementary Table 2. Performance of tests in the entire cohort (including 95% CI).

Supplementary Table 3. Performance of tests in the subgroup of adrenal masses with unenhanced HU > 10 (including 95% CI).

Supplementary Table 4a. Baseline characteristics of subgroup of patients with incidentally detected tumors. b. Performance of tests in the subgroup of patients with incidentally detected tumors (n=174).

Supplementary Table 5a . Baseline characteristics of patients with homogenous adrenal lesions in unenhanced CT. b. Performance of tests in the subgroup of patients with homogenous adrenal lesions in unenhanced CT.

## Declaration of interest

Martin Fassnacht is a senior editor of the *European Journal of Endocrinology* and was not involved in the peer review or editorial process for this paper on which he is listed as an author. The other authors declare no competing interest.

## Funding

This study was supported in part by the Deutsche Forschungsgemeinschaft http://dx.doi.org/10.13039/501100001659 (DFG) project number 314061271 – TRR 205 to M F.
