# Supplementary material for: Adrenal wash-out CT: moderate diagnostic value in distinguishing benign from malignant adrenal masses
Source: Eur J Endocrinol. 2021 Nov 23;186(2):183–93. doi: 10.1530/EJE-21-0650 (PMC8679842; doi:10.1530/EJE-21-0650)
Supplement: Supplementary Table 1. Criteria to categorize the different adrenal masses and numbers per groups. [file supplementary_table_1.pdf]

Supplementary Table 1. Criteria to categorize the different adrenal masses and numbers per groups.

**Criteria**	No. (%)
Benign adrenal mass	
• Histopathological confirmation	61 (24.2)
• Size stability >12 months	101 (40.1)
• Size stability >6 months and FDG-PET negative	3 (1.2)
• Calculated growth of < 10% or < 1 mm per year in cases of long-term follow-up[1]	20 (7.9)
• No clinical sign of malignant disease > 5 years	18 (7.1)
Malignant adrenal masses	
• Histopathological confirmation	31 (12.3)
• Progression in size >20% or > 1 cm per year paralleled by progressive or newly appeared tumoral lesions indicating progressive disease	6 (2.4)
• Progression in size of ≥ 100 % and ≥ 1,5 cm within one year	0
• Progression in size of at least 10 % or 1cm per year and strong evidence of malignancy by FDG-PET	0
• Regression in size of the adrenal mass in cases of a general response of the malignancy to a tumor-specific therapy	7 (2.8)
• New adrenal lesion with size of ≥ 2 cm in a patient with a history of extra-adrenal malignancy	3 (1.2)
• New adrenal lesion in a patient with a history of extra-adrenal malignancy and strong evidence of malignancy by FDG-PET	0
• New adrenal lesion in a patient with a history of extra-adrenal malignancy and other progressive or newly appeared tumoral lesions	2 (0.8)
**Total**	*252 (100)*

[1] defined as imaging later than 5 years after the baseline CT
