# Supplementary material for: Adrenal wash-out CT: moderate diagnostic value in distinguishing benign from malignant adrenal masses
Source: Eur J Endocrinol. 2021 Nov 23;186(2):183–93. doi: 10.1530/EJE-21-0650 (PMC8679842; doi:10.1530/EJE-21-0650)
Supplement: Supplementary Table 2. Performance of tests in the entire cohort (including 95% CI). [file supplementary_table_2.pdf]

Supplementary Table 2. Performance of tests in the entire cohort (including 95% CI).

Test categories	Cutoff	Benign (n=203)	(Potentially) malignant (n=49)	% of benign cases	% of (potentially) malignant cases	PPV % (95% CI)	NPV % (95% CI)
**Tumor size**							
	< 4cm	159	22	78.3 (72.2-83.4) [1]	44.9 (31.9-58.7)	87.9 (84.0 – 90.9)	38.0 (30.0 – 46.9)
	≥ 4cm	44	27	21.7 (16.6-27.8)	55.1 (41.3-68.1) [2]		
**Unenhanced Hounsfield Units**							
	≤ 10	110	0	54.2 (47.3-60.9) [1]	0 (0-7.3)	100	34.5 (31.2 – 38.0)
	> 10	93	49	45.8 (39.1-52.7)	100 (92.7-100) [2]		
**Absolute percentage wash-out**							
	> 60%	130	11	64.0 (57.2-70.3) [1]	22.4 (13.0-35.9)	92.2 (87.4 – 95.3)	34.2 (29.1 – 39.8)
	≤ 60%	73	38	36.0 (29.7-42.8)	77.6 (64.1-87.0) [2]		
	> 83%	17	1	8.4 (5.3-13.0) [1]	2.0 (0.1-10.7)	94.4 (69.9 – 99.2)	20.5 (19.6 – 21.5)
	≤ 83%	186	48	91.6 (87.0-94.7)	98.0 (89.3-99.9) [2]		
**Relative percentage wash-out**							
	> 40%	140	4	69.0 (62.3-74.9) [1]	8.2 (3.2-19.2)	97.2 (93.2 – 98.9)	41.7 (36.4 – 47.1)
	≤ 40%	63	45	31.0 (25.1-37.7)	91.8 (80.8-96.8) [2]		
	> 58%	93	1	45.8 (39.1-52.7) [1]	2.0 (0.1-10.7)	98.9 (93.0 – 99.9)	30.4 (27.7 – 33.3)
	≤ 58%	110	48	54.2 (47.3-60.9)	98.0 (89.3-99.9) [2]		

[1] Sensitivity

[2] Specificity
