# Supplementary material for: Adrenal wash-out CT: moderate diagnostic value in distinguishing benign from malignant adrenal masses
Source: Eur J Endocrinol. 2021 Nov 23;186(2):183–93. doi: 10.1530/EJE-21-0650 (PMC8679842; doi:10.1530/EJE-21-0650)
Supplement: Supplementary Table 3. Performance of tests in the subgroup of adrenal masses with unenhanced HU > 10 (including 95% CI). [file supplementary_table_3.pdf]

Supplementary Table 3. Performance of tests in the subgroup of adrenal masses with unenhanced HU > 10 (including 95% CI).

Test categories	Cutoff	Benign (n=93)	(Potentially) malignant (n=49)	% of benign cases	% of (potentially) malignant cases	PPV % (95% CI)	NPV % (95% CI)
**<u>Tumor size</u>**							
	< 4cm	70	22	75.3 (65.2-83.6) [1]	44.9 (30.7-59.8)	76.1 (69.6-81.6)	54.0 (43.2-64.5)
	≥ 4cm	23	27	24.7 (16.4-34.8)	55.1 (40.2-69.3) [2]		
**<u>Absolute percentage wash-out</u>**							
	> 60%	53	11	57.0 (46.3-67.2) [1]	22.4 (11.8-36.6)	82.8 (73.6-89.3)	48.7 (41.8-55.7)
	≤ 60%	40	38	43.0 (32.8-53.7)	77.6 (63.4-88.2) [2]		
	> 83%	10	1	10.8 (5.3-18.9) [1]	2.0 (0.1-10.9)	90.9 (56.9-98.7)	36.6 (34.8-38.6)
	≤ 83%	83	48	89.2 (81.1-94.7)	98.0 (89.1-99.9) [2]		
**- Without pheos**	**> 83%**	**10**	**1**	**10.8 (5.3-18.9)** [1]	**2.6 (0.1-13.8)**	**90.9 (57.0-98.7)**	**30.8 (29.0-32.7)**
**(n=131)**	**≤ 83%**	**83**	**37**	**89.2 (81.1-94.7)**	**97.4 (86.2-99.9)** [2]		
**<u>Relative percentage wash-out</u>**							
	> 40%	47	4	50.5 (40.0-61.1) [1]	8.2 (2.3-19.6)	92.2 (81.8-96.9)	49. 5 (43.9-55.0)
	≤ 40%	46	45	49.5 (38.9-60.0)	91.8 (80.4-97.7) [2]		
	> 58%	14	1	15.1 (8.5-24.0) [1]	2.0 (0.1-10.9)	93.3 (65.5-99.0)	37.8 (35.6-99.0)
	≤ 58%	79	48	84.9 (76.0-91.5)	98.0 (89.1-99.9) [2]		
**- Without pheos**	**> 58%**	**14**	**0**	**15.1 (8.5-24.0)** [1]	**0 (0-9.3)**	**100**	**32.5 (30.6-34.4)**
**(n=131)**	**≤ 58%**	**79**	**38**	**84.9 (76.0-91.5)**	**100 (90.7-100)** [2]		

[1] Sensitivity

[2] Specificity;

Pheos pheochromocytomas
