# Supplementary material for: Adrenal wash-out CT: moderate diagnostic value in distinguishing benign from malignant adrenal masses
Source: Eur J Endocrinol. 2021 Nov 23;186(2):183–93. doi: 10.1530/EJE-21-0650 (PMC8679842; doi:10.1530/EJE-21-0650)
Supplement: Supplementary Table 4a. Baseline characteristics of subgroup of patients with incidentally detected tumors. b. Performance of tests in the subgroup of patients with incidentally detected tumors (n=174). [file supplementary_table_4.pdf]

Supplementary Table 4a. Baseline characteristics of subgroup of patients with incidentally detected tumors.

	Entire cohort	Subgroup with adrenal mass HU > 10 in unenhanced CT
**Patients, no.**	145	84
**Sex, no. (%)**		
Male	80 (55)	48 (57)
Median age (range)	59 (32-83)	59 (32-82)
**Side of adrenal mass, no**	(% of 145 patients)	(% of 96 patients)
Right	44 (30.3)	34 (35.4)
Left	73 (50.4)	50 (52.1)
Bilateral	28 (19.3)	12 (12.5)
**Total number of adrenal masses**	**174**	**96**
**Final diagnosis**		
Benign	145	67
Adrenal adenoma/ hyperplasia	117	51
Other benign tumor	28	16
Malignant	20	20
Metastasis	13	13
Adrenocortical carcinoma	5	5
Lymphoma	2	2
Potentially malignant (Pheos)	9	9

Supplementary Table 4b. Performance of tests in the subgroup of patients with incidentally detected tumors (n=174).

Test categories	Cutoff	Benign (n=145)	(Potentially) malignant (n=29)	% of benign cases (95% CI)	% of (potentially) malignant cases (95% CI)
**Tumor size**					
	< 4cm	107	10	73.8 (65.8-80.7) [1]	34.5 (17.9-54.3)
	≥ 4cm	38	19	26.2 (19.3-34.2)	65.5 (45.7-82.1) [2]
**Unenhanced Hounsfield Units**					
	≤ 10	78	0	53.8 (45.3-62.1) [1]	0 (0-11.9)
	> 10	67	29	46.2 (37.9-54.7)	100 (88.1-100) [2]
**Absolute percentage wash-out**					
	> 60%	84	7	57.9 (49.5-66.1) [1]	24.1 (10.3-43.4)
	≤ 60%	61	22	42.1 (33.9-50.5)	75.9 (56.5-89.7) [2]
	> 83%	12	1	8.3 (4.4-14.0) [1]	3.5 (0.01-17.8)
	≤ 83%	133	28	91.7 (86.0-95.6)	96.5 (82.2-99.9)
without pheos (n=9)	> 83%	12	1	8.3 (4.4-14.0) [1]	5.0 (0.1-17.8)
	≤ 83%	133	19	91.7 (86.0-95.6)	95.0 (75.1-99.9) [2]
**Relative percentage wash-out**					
	> 40%	91	2	62.8 (54.4-70.6) [1]	6.9 (0.8-22.8)
	≤ 40%	54	27	37.2 (29.4-45.6)	93.1 (77.2-99.2) [2]
	> 58%	58	0	40.0 (32.0-48.5) [1]	0.0 (0.0-11.9)
	≤ 58%	87	29	60.0 (51.5-68)	100.0 (88.1-100.0) [2]
without pheos	> 58%	58	0	40.0 (32.0-48.5) [1]	0.0 (0.0-16.8)
(n=165)	≤ 58%	87	20	60.0 (51.5-68)	100.0 (83.2-100.0) [2]

[1] Sensitivity

[2] Specificity;

Pheos pheochromocytomas
