# Supplementary material for: Adrenal wash-out CT: moderate diagnostic value in distinguishing benign from malignant adrenal masses
Source: Eur J Endocrinol. 2021 Nov 23;186(2):183–93. doi: 10.1530/EJE-21-0650 (PMC8679842; doi:10.1530/EJE-21-0650)
Supplement: Supplementary Table 5a . Baseline characteristics of patients with homogenous adrenal lesions in unenhanced CT. b. Performance of tests in the subgroup of patients with homogenous adrenal lesions in unenhanced CT. [file supplementary_table_5.pdf]

Supplementary Table 5a . Baseline characteristics of patients with homogenous adrenal lesions in unenhanced CT.

	Entire cohort	Subgroup with adrenal mass HU > 10 in unenhanced CT
**Patients, no.**	144	85
**Sex, no. (%)**		
Male	80 (56)	50 (59)
Median age (range)	64 (30-83)	71 (30-83)
**Mode of detection of the adrenal mass (%)**		
Incidental finding	107 (62.6)	57 (60)
Suspected adrenal disease	24 (14)	10 (10.5)
Tumor evaluation due to an extra-adrenal malignancy	40 (23.4)	28 (24.2)
**Side of adrenal mass, no**	(% of 144 patients)	(% of 85 patients)
Right	36 (25)	25 (29.4)
Left	81 (56.3)	50 (58.8)
Bilateral	27 (18.7)	10 (11.8)
**Total number of adrenal masses**	**171**	**95**
**Final diagnosis**[1]		
Benign	136	60
Adrenal adenoma/ hyperplasia	121[2]	49[3]
Other benign tumor	15[4]	11[5]
Malignant	31	31
Metastasis	20[6]	20[6]
Adrenocortical carcinoma	7[7]	7[7]
Lymphoma	4[8]	4[8]
Potentially malignant	4	4
Pheochromocytoma	4[7]	4[7]

[1] for definition, see Supplementary Table 1
[2] 24 confirmed by histology
[3] 14 confirmed by histology
[4] 5 confirmed by histology: including histological-confirmed atypical myelolipomas (n=2), atypical adrenal cysts (n=1), ganglioneuromas (n=1), tuberculosis-related adrenal masses (n=1).
[5] 3 confirmed by histology: including histological-confirmed atypical adrenal cysts (n=1), ganglioneuromas (n=1), tuberculosis-related adrenal masses (n=1).
[6] including primaries from urogenital system (n=4), skin (n=4), lung (n=2), gastrointestinal system (n=4), breast (n=1), leiomyosarcoma (n=2), liver (n=1), breast (n=1), CUP (n=1), etc.; among them, 6 were confirmed by histology
[7] all confirmed by histology
[8] one confirmed by histology

Supplementary Table 5b. Performance of tests in the subgroup of patients with homogenous adrenal lesions in unenhanced CT.

**Test categories**	**Cutoff**	**Benign (n=136)**	**(Potentially) malignant (n=35)**	**% of benign cases (95% CI)**	**% of (potentially) malignant cases (95% CI)**
**Tumor size**					
	< 4cm	120	18	88.2 (81.6-93.1) [1]	51.4 (34.0-68.6)
	≥ 4cm	16	17	11.8 (6.9-18.4)	48.6 (31.4-66)[2]
**Unenhanced Hounsfield Units**					
	≤ 10	76	0	55.9 (47.1-64.4)[1]	0 (0-10)
	> 10	60	35	44.1 (35.6-52.9)	100 (90-100)[2]
**Absolute percentage wash-out**					
	> 60%	101	9	74.3 (66.1-81.4) [1]	25.7 (12.5-43.3)
	≤ 60%	35	26	25.7 (18.6-33.9)	74.3 (56.7-87.5) [2]
	> 83%	13	1	9.6 (5.2-15.8) [1]	2.9 (0.1-14.9)
	≤ 83%	123	34	90.4 (84.2-94.8)	97.1 (85.1-99.9) [2]
**Without pheos**	**> 83%**	**13**	**1**	**9.6 (5.2-15.8) [1]**	**3.2 (0.1-16.7)**
**(n=167)**	**≤ 83%**	**123**	**30**	**90.4 (84.2-94.8)**	**96.8 (83.3-99.9) [2]**
**Relative percentage wash-out**					
	> 40%	110	3	80.9 (73.6-87.1) [1]	8.6 (1.8-23.1)
	≤ 40%	26	32	19.1 (12.9-26.4)	91.4 (76.9-98.2) [2]
	> 58%	74	0	54.4 (45.7-63.0) [1]	0 (0-10.0)
	≤ 58%	62	35	45.6 (37-54.3)	100 (90.0-100.0) [2]
**Without pheos**	**> 58%**	**74**	**0**	**54.4 (45.7-63.0) [1]**	**0 (0-11.2)**
**(n=167)**	**≤ 58%**	**62**	**31**	**45.6 (37-54.3)**	**100 (88.8-100.0) [2]**

[1] Sensitivity
[2] Specificity;
Pheos pheochromocytomas
